# A Window of Opportunity: Radical Versus Repurposing Innovation Under Conditions of Environmental Uncertainty and Crisis

**DOI:** 10.1109/tem.2023.3282803

**Published:** 2023-06-16

**Authors:** Denise R. Dunlap, Roberto S. Santos, Scott F. Latham

**Affiliations:** Department of Marketing, Entrepreneurship and Innovation and the Department of Management, Manning School of Business, University of Massachusetts Lowell, Lowell, MA 01854 USA; Girard School of Business, Merrimack College, North Andover, MA 01848 USA; Department of Marketing, Entrepreneurship and Innovation and the Department of Management, Manning School of Business, University of Massachusetts Lowell, Lowell, MA 01854 USA

**Keywords:** COVID-19, crisis, knowledge, radical innovation, repurposed innovation

## Abstract

In this article, we extend the innovation literature by examining how firms respond to crisis, specifically exogenous crises. At their early onset, crises may represent a window of opportunity for innovation, but it is not equally allocated across firms. We created a unique database of 636 biopharmaceutical firms, from 24 countries and territories, developing innovative treatments during the early outbreak of the COVID-19 crisis to study this phenomenon. We found that firms acted strategically to the shifting external environment and attempted to capitalize on the opportunity by pursuing different but complementary innovation strategies (i.e., radical versus repurposed). The successful outcome of a chosen strategy was highly dependent upon a firm’s accumulated knowledge resources, which varied in degree of diversity (i.e., homogeneous versus heterogeneous). We found that firms with more focused R&D (i.e., homogeneous knowledge) developed more radical innovations, whereas firms with more diverse R&D (i.e., heterogeneous knowledge) repurposed innovations. We controlled for firm size (small versus large), firm age (startup versus mature), and country classification (developing versus emerging). We also controlled for a firm’s prior knowledge and expertise in coronavirus research and found that it did not influence innovation. Our results suggest that this unique period of environmental uncertainty and crisis created a window of opportunity and a level playing field for innovation.

## I. Introduction

“If you look at three diseases, the three major killers, HIV, tuberculosis, and malaria, the only disease for which we have really good drugs is HIV. And it is very simple: because there is a market in the United States and Europe.”

– J. Y. Kim, Former President of the World Bank

In 2018, epidemics cost the world $60 billion yearly [1]. The 2019 National Institutes of Health (NIH) budget was $39 billion [2], with 16% of funding allocated toward infectious diseases, predominately HIV (or 48.1%) [3], [4]. The anti-infective therapeutic research area, at the end of 2020, was in decline [5]. The global nature of the coronavirus pandemic questioned many taken-for-granted assumptions about our understanding of infectious diseases and our ability to eradicate them. It was one of those rare exogenous shocks that was difficult to predict and, unlike internal crises (e.g., Tylenol crisis of 1982), was largely out of the firm’s control [6]. Yet, once it unfolded, the magnitude of its related disruption was far-reaching and perpetually driven by a rise in viral strain mutations, resulting in an estimated impact of $16 trillion [7].

While this was not the first global pandemic that we, as a society, have confronted (e.g., the 1918 “Spanish Flu” outbreak), there were prior close calls with other infectious diseases (e.g., SARS, MERS, and Ebola). For far too long, the nature and spread of contagious diseases (e.g., Ebola) have been predominantly confined to the poorest parts of the world. Investment in infectious disease research, including vaccines, has been largely unattractive to most firms since there are often negligible probabilities of recovering high investment costs (e.g., [8]). Whether due to cognitive myopia, political exigency, or emotional insensitivity, such perpetuating attitudes contributed to nations finding themselves unprepared to respond to this environmental health crisis [9].

While this period of disruption challenged firms’ *modus operandi,* it also inadvertently opened a window of opportunity for new entrants and technological advances. Currently, there remains a dearth of research regarding how firms strategically respond to the innovation demands posed by a crisis, and more specifically a health crisis (e.g., [10], [11], [12]). These unique periods of historical crisis remain an increasingly important area of scholarly inquiry in the innovation and crisis management literature works [12], [13], especially for research and knowledge intensive industries, which are traditionally characterized by high uncertainty and low probabilities of innovation success (e.g., [14]). In our study, we seek to address the gap in the literature by addressing the following question: *When an exogenous crisis first emerges, how does the organization’s existing knowledge stocks affect its ability to capitalize on the opportunity through innovation?*

The existing literature about the relationship between knowledge and innovation has been mainly concentrated on studies conducted during periods of noncrisis (e.g., [15], [16]), especially in environments of relative certainty or technological change (i.e., relative uncertainty). In this context, heterogeneous knowledge (i.e., diverse knowledge) is often associated with improving the odds of developing radical innovations since it increases the number of different elements that can be combined to create radical innovations (e.g., [17], [18]). Regarding the development of incremental improvements, including repurposing innovations, the literature has also found that possessing more homogeneous knowledge (i.e., focused knowledge) is beneficial [19], [20], [21]. Our study seeks to develop a better understanding about the relationship between accumulated knowledge stocks, which vary in degree of diversity (i.e., homogeneous versus heterogeneous) (e.g., [22]), and innovation (radical versus repurposed) in the context of a crisis and whether this represents an opportunity for firms.

To address our inquiry, we created a novel dataset that included 636 biopharmaceutical firms from 24 countries and territories working on innovative treatments to combat the coronavirus. Our study focuses on the peak period of uncertainty before the first commercially available vaccines came to market. We find strong evidence that a firm’s prior know-how played a significant role in how it targeted its R&D toward developing new therapeutics. Firms with more diverse R&D (i.e., heterogeneous knowledge) were likelier to repurpose existing innovations rather than create radical ones. Conversely, those firms with more focused R&D (i.e., homogeneous knowledge) developed more radical innovations in the early stages of the pandemic. Further, our findings indicate that this period of uncertainty created more equality across firms (small versus large and startup versus mature) and countries (developing versus emerging) within a highly competitive industry. Interestingly, initial investment in coronavirus research did not provide a competitive advantage for our studied firms.

Our study contributes to the innovation and crisis management literature works by broadening our understanding about how exogenous crises may offer opportunities for firms that are able to recognize these important shifts in the external environment. Our study extends the conversation about how an organization’s accumulated knowledge resources (heterogeneous versus homogeneous knowledge) influences its response strategy to innovation (radical versus repurposed). Our findings offer a nuanced perspective about the relationship between knowledge and innovation in times of crisis. Finally, our study makes important contributions to the international business literature by revealing new insights about how firms from different countries approached innovation challenges during an exogenous crisis.

The rest of this article is structured as follows. In Section II, we review the relevant literature and present our hypotheses. We describe our methodological approach in Section III and present our empirical results in Section IV. We discuss the relevance of our findings and theoretical and practical implications in Section V and state our conclusions in Section VI.

## II. Literature Review and Hypothesis Development

### A. Exogenous Crisis and Innovation

In their review of the literature, Cameron et al. [23] suggest that environmental discontinuities (i.e., crises, turbulence) create disruptions outside of the firm’s control and have significant consequences on its associated response strategies. Williams and colleagues [24] view a crisis as a process that weakens and disrupts the normal functioning of organizations, which may influence firm survival (e.g., [25], [26]). We define a crisis, specifically exogenous crisis, across several dimensions. An exogenous crisis initially results from a larger exogenous shock (e.g., recession, war, pandemic). It emerges unexpectedly and is largely unpredictable. Most importantly, an exogenous crisis presents a dichotomy relative to the nature of the event. It can represent a significant threat to the firm or present a window of opportunity. Firms capable of recognizing shifts in the external environment, resulting from the crisis, must weigh their opportunity costs. They may choose to invest in repositioning costs (i.e., acquired knowledge, assets, and capabilities) needed to respond to the emerging and new-post shock conditions. Successful firms strategically reposition themselves to be able to capitalize upon these innovative shock opportunities [27].

Currently, there is a growing inquiry among scholars that want to develop a deeper understanding about how firms build resilience in the aftermath of a crisis (e.g., [24], [28]), including financial, economic, and natural disasters and more recently, doomsday scenarios involving virulent pathogens (e.g., [9], [29]). These crisis-based studies (e.g., [30], [31], [32], [33], [34]) are increasingly interested in examining how crises foster entrepreneurship (e.g., [13], [35], [36], [37], [38]) and innovation (e.g., [12], [39], [40], [41], [42], [43]) across different stakeholders in the innovation ecosystem [44], [45]. Given more recent events (i.e., the coronavirus pandemic), scholars acknowledge that crises continue to alter our fundamental assumptions and theories about innovation and technology management and, thus, necessitates future ongoing discussions and debates [12]. Table I provides a synthesis of the emerging and evolving body of crisis-based research in the context of novel contributions to the innovation literature.

Innovation is at the heart of a firm’s sustained competitive advantage [46]. It is fraught with uncertainties and introduces considerable risks to the firm [47]. As an inherently risky endeavor, innovation involves committing substantial resources (e.g., experimentation, intellectual property protection, and human capital) with no promise of success, even when focused on the most lucrative markets (e.g., oncology versus infectious disease research). Even if the payback from such R&D expenditures is uncertain, Bowman and Hurry [48] put forth an option-theoretic perspective that organizations are motivated to innovate while simultaneously “keeping options open” to mitigate unforeseeable risks. Innovation is consequently a chance worth pursuing to capitalize on a potential opportunity embedded in a crisis.

When faced with an unpredictable, high-profile crisis, nations often turn to science for innovative solutions [49]. Addressing a crisis requires new behaviors, new technology, and new products, in other words, innovation. However, whereas the characterization of such activities may appear straightforward at first glance, the nature of innovating in a crisis is far more complex. First, the pace of innovation changes. For instance, as Gross and Sampat [49] argue, innovation during crises and noncrises differs in that speed is one of the primary objectives during a crisis. Such predicaments accentuate the need for successful and timely innovations [34], [43], with some firms accruing first-mover advantages (e.g., [50]). Second, recognizing that innovation is risky, even under the best circumstances, it is worth noting that innovative endeavors are further exacerbated within the larger context of a crisis (e.g., supply chain disruptions, labor shortages, and damaged infrastructure) [51]. Hence, since exogenous problems are marked by a high degree of ambiguity, there is also a heightened risk of failure. Finally, and by extension, even the most all-encompassing strategies have unintended short- and long-term impacts (e.g., stock price fluctuations, plant closures, employee layoffs). As Cameron and colleagues [52] acknowledge, implementing the wrong strategy, poorly executing a well-planned strategy, or having no strategy at all can have devastating consequences for firms and their limited resources. If a firm pursues innovation and it subsequently fails, then its associated resources will have been lost and may impact its performance (e.g., [53], [54], [55]) or viability (e.g., [25]). Consequently, it is imperative to explore the strategies firms employ to innovate during a crisis scenario, which demands timely solutions with unforeseen outcomes.

On the one hand, firms may respond to crises by strategically husbanding their scarce resources and curtailing R&D activities [56], [57]. On the other hand, firms that see crises as opportunities must weigh their innovative options [57]. The extant literature has described the advantages these firms may accrue when entering a new market, including capturing market share, building brand recognition, and attracting a loyal customer following [50], [58]. Indeed, firms may be motivated to enter markets early to secure a competitive position [59]. Doing so may involve different innovation strategies [60], such as developing novel innovations (i.e., radical), repurposing existing innovations (i.e., incremental), or doing some combination of both. Revolutionary innovations, such as radical ones, are generally considered new-to-the-world, whereas incremental innovations comprise various improvements to existing technologies. In instances where a radical innovation has yet to be discovered, often in a crisis, the most expedient option is frequently to repurpose a current invention. Repurposed innovations are a unique subset of incremental innovations and are also the focus of our inquiry.

The successful outcome of a chosen innovation strategy (radical versus repurposed) is highly dependent upon a firm’s accumulated knowledge stocks, which vary in their degree of diversity (i.e., homogeneous versus heterogeneous) (e.g., [22]). Given the lengthy nature of the innovation process, coupled with the infrequency of unforeseen crisis events, much of the scholarly inquiry about the relationship between knowledge and innovation, up until recently, has been focused on periods of non-crisis (e.g., [15], [16]). Recognizing that most crises are rare events, with most occurring locally (e.g., San Francisco Plague, Ebola) and even fewer reaching a global magnitude (e.g., Black Death, Spanish flu), it is critical to study the strategic relationship between firm knowledge stocks and innovation under these circumstances.

### B. Knowledge and Innovation

**Fig. 1.**
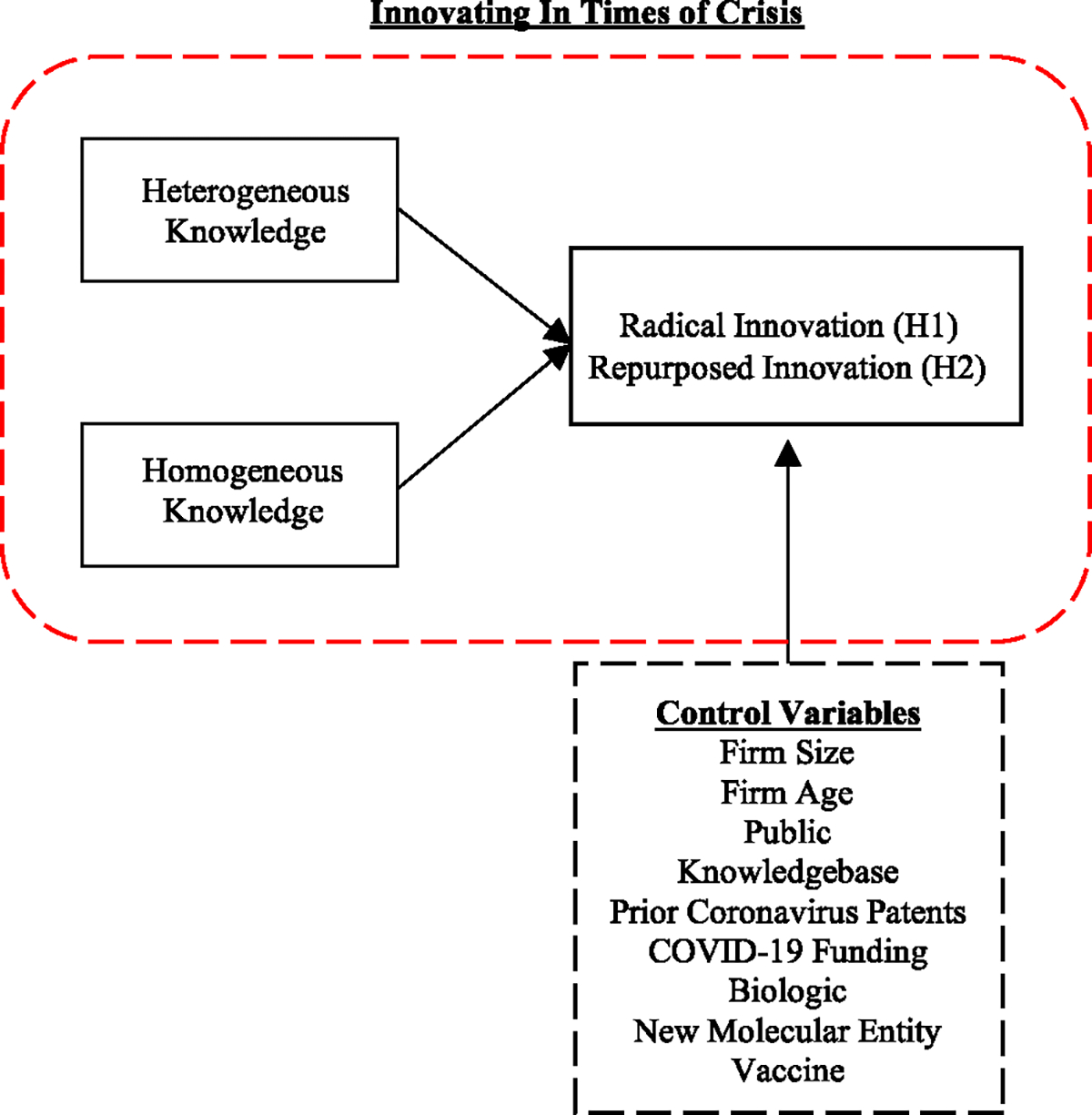
Conceptual model.

Knowledge has long been regarded as a valuable resource that can provide firms with a sustained competitive advantage [61]. When innovating, firms, particularly high-tech firms, rely upon and combine different knowledge elements (e.g., [17]), which may be heterogeneous or homogeneous in nature. On the one hand, heterogeneity of knowledge, or what others have described as the breadth of expertise, refers to the diversity of a firm’s accumulated knowledge stocks [19], [62]. On the other hand, homogeneous knowledge is recognized among scholars as encompassing deep expertise in a specialized field or subject domain [19]. Our study builds upon the overarching notion that firms typically accumulate one type of knowledge stock over another (i.e., homogeneous versus heterogeneous) and, due to sunk costs, it may preclude firms from pursuing alternative opportunities to innovate outside of their area(s) of expertise (e.g., [18]), particularly in a crisis scenario. To this end, we conjecture that during these periods of immense uncertainty, building upon one’s existing knowledge stocks may be more efficient for innovation (i.e., radical versus repurposed) (see Fig. 1 for our conceptual model). Strategically knowing how to use one’s knowledge in a crisis may facilitate results in timely solutions.

#### 1) Radical Innovation:

Radical innovations are novel ideas or products that stem from creating new knowledge [63]. Despite being costly and time-consuming endeavors with uncertain outcomes, they have long been of scholarly interest because they have the power to create new markets (e.g., [64], [65]). Even though the process by which radical innovations are created is complex and accompanied by a low probability of success, doing so is often necessary for firms to survive in competitive environments (e.g., [64]). Among those firms that commercialize these innovations successfully, their ability to tap into heterogeneous pools of knowledge across the organization was vital to their success (e.g., [18], [66]).

Although the process of producing radical innovations is ever uncertain, what the existing literature espouses under periods of noncrisis is that firms with a more significant stock of heterogeneous knowledge expertise have a greater quantity of unique knowledge elements that can be reconfigured and combined to produce ground-breaking ideas [17], [18], [20], [67]. Radical innovations, such as penicillin, the light bulb, steam engine, nuclear fission, the Internet, smart phones, and cloud computing, among others, have all come about due to the combination of diverse knowledge elements. A recent illustrative example involves the introduction of the talking hearing aid that integrates acoustics, artificial intelligence, sensors, and electronics knowledge. Another technology, decades in the making, that has seen recent advances in the laboratory is nuclear fusion, which is made possible by combining knowledge from diverse areas including physics, photonics, materials science, and electronics. As these scenarios indicate, the timeframe from idea generation to commercialization involves years, if not decades, of dedicated research involving multiple domains of expertise. We argue that under periods of crisis, such long timeframes are a luxury that radical innovation cannot afford.

While it is well-established that possessing greater knowledge diversity facilitates the generation of radical innovations, it remains a significant and daunting endeavor to form mental linkages across disparate knowledge elements, even under the best-case scenarios, due to rigidities, path-dependences, and cognitive biases (e.g., [68], [69]). Accordingly, when faced with greater time constraints, such as those imposed by a crisis, a firm’s ability to apply its heterogeneous knowledge stocks toward radical innovations may be further and significantly challenged (i.e., high coordination costs, resource disruptions) [19], [70]. Herein, we contend that focus and deep expertise, related to the crisis at hand, may yield more positive outcomes for radical innovation than heterogeneous knowledge.

In this regard, firms with deep expertise, especially if it falls within a common domain, have an enhanced ability to harness their interrelated knowledge stocks towards creating novel solutions with greater ease and efficiency [19], [20], [71], which is required in a crisis. Building knowledge expertise of this kind requires years of focused R&D investment with huge sunk costs (e.g., plant facilities, capital investments, equipment, human resources, etc.). During this time, firms develop a shared cognitive understanding, which facilitates mastery of a particular domain (e.g., [18], [68], [69]).

If a firm’s accumulated knowledge stock is associated with a particular specialty, then the firm may have an increased competence to know how to filter out lower quality ideas quickly so as not to divert scarce resources needed for the development of radical innovations (e.g., [19]). In times of great crisis, when time is of the essence, expertise and deep knowledge are valuable. For instance, during World War II, A. Turing, a British codebreaker, used his deep knowledge of mathematics to crack the German Enigma code, which was a crucial turning point in the war, resulting in an estimated 14–21 million lives saved and a shortening of the war by two to three years [72]. This is one example that demonstrates the profound impact that homogeneous knowledge, wielded by experts, can have on radical innovations needed in crises. These revolutionary innovations often impact an industry well beyond the crisis (e.g., Turing’s innovations formed the foundation of modern computing and artificial intelligence). Based on this rationale, we argue that in times of crisis, homogeneous knowledge may foster radical innovations to a greater extent. Hence, we hypothesize the following.

H1: During periods of crisis, firms with greater homogeneous knowledge will develop radical innovations to a greater extent than firms with heterogeneous knowledge.

#### 2) Repurposing Innovation:

While developing radical innovations is one approach to addressing a crisis, another option is repurposing or reusing existing technologies in a different context [43]. This strategy can be effective when economic incentives for developing of new technologies are absent [73] or when valuable resources are scarce [46]. Although repurposed innovations are often perceived as less innovative, they are not necessarily inferior and may offer comparable performance to radical innovations when expediency is necessary (e.g., [73]). Some additional benefits of repurposing include allowing the firm to do more with fewer resources and to do so quickly, affordably, and efficiently in times of great need and urgency (e.g., [74]). Prior studies have shown that repurposing innovations can have great value (e.g., [75]) when existing solutions are unavailable, and the cost is paramount [43], [76]. Moreover, repurposing has proven to be a particularly effective innovation strategy for treating rare diseases [73].

There appears to be some agreement about the benefits of repurposing innovation (e.g., [43], [73], [76]). Although the literature on this topic is still evolving, it would be advantageous to understand how firms draw upon their knowledge stocks to repurpose innovations (e.g., [73]). The literature has primarily studied all types of incremental innovations as one category and typically has yet to differentiate among various kinds, including repurposed innovations (e.g., [22]). The scholarly consensus has been that having more niche specific, homogeneous know-how leads to the development of incremental innovations during periods of certainty (e.g., [77]). An illustrative example of a firm that has successfully used this strategy is Abbvie, a biopharmaceutical firm. Over two decades, the firm was able to capitalize upon its homogeneous knowledge of inflammatory disorders to create multiple incremental improvements to its existing blockbuster drug, Humira (e.g., dosage, delivery mechanism, removing citrate buffers). This know-how further allowed the firm to use drug repurposing to treat other ailments (e.g., rheumatoid arthritis, plaque psoriasis, ankylosing spondylitis, Crohn’s disease, and ulcerative colitis). *Ceteris paribus*, we acknowledge that in periods of relative certainty, firms such as these may prefer to exploit their homogeneous knowledge stocks to these ends because it is cognitively efficient (e.g., [18]).

Under periods of uncertainty, however, there may be a mismatch between what the firm already possesses (i.e., a firm’s homogeneous knowledge may fall outside of the domain of expertise needed) and what is required to find an expedient solution. Herein, we purport that, during crises, the comingling of knowledge stocks from multiple domains may be advantageous for identifying opportunities to repurpose innovations. Relying upon this strategy may be particularly effective in periods of profound magnitude (e.g., war, pandemics, etc.) where resourcefulness and ingenuity are necessary. For instance, before the outbreak of World War II, parachutes and hosiery were traditionally made of silk, which was sourced from Asia. However, due to the onset of the war, silk exports quickly evaporated, creating a crisis for military pilots and paratroopers. Drawing upon its heterogeneous knowledge of chemistry, materials science, manufacturing processes, and textiles, Dupont was able to repurpose its synthetic fiber nylon, which was initially used to make stockings (i.e., nylons), toward the production of parachutes and parachute cords during wartime. This repurposed innovation has had an untold impact on the industry’s growth. Nylon became the *de facto* standard for parachutes, among other military and domestic applications, and continues to be used today (e.g., toothbrushes, athletic apparel).

Building upon examples such as these, we argue that periods of uncertainty may enhance opportunity recognition by alleviating some of the barriers that traditionally create cognitive myopia across organizations (e.g., silos). In this sense, firms with heterogeneous knowledge stocks, compared to firms with more niche-specific know-how (i.e., homogeneous), may be better poised to recognize opportunities that match the diversity of knowledge in their possession, particularly in times of significant technological and scientific turbulence (e.g., [18]). We argue that finding a desirable solution in a crisis, via the repurposing of innovations, may require a more heterogeneous approach. Accordingly, we hypothesize the following.

H2: During periods of crisis, firms with greater heterogeneous knowledge will repurpose innovations to a greater extent than firms with homogeneous knowledge.

### III. Methods

#### A. Sample and Data Sources

**Fig. 2.**
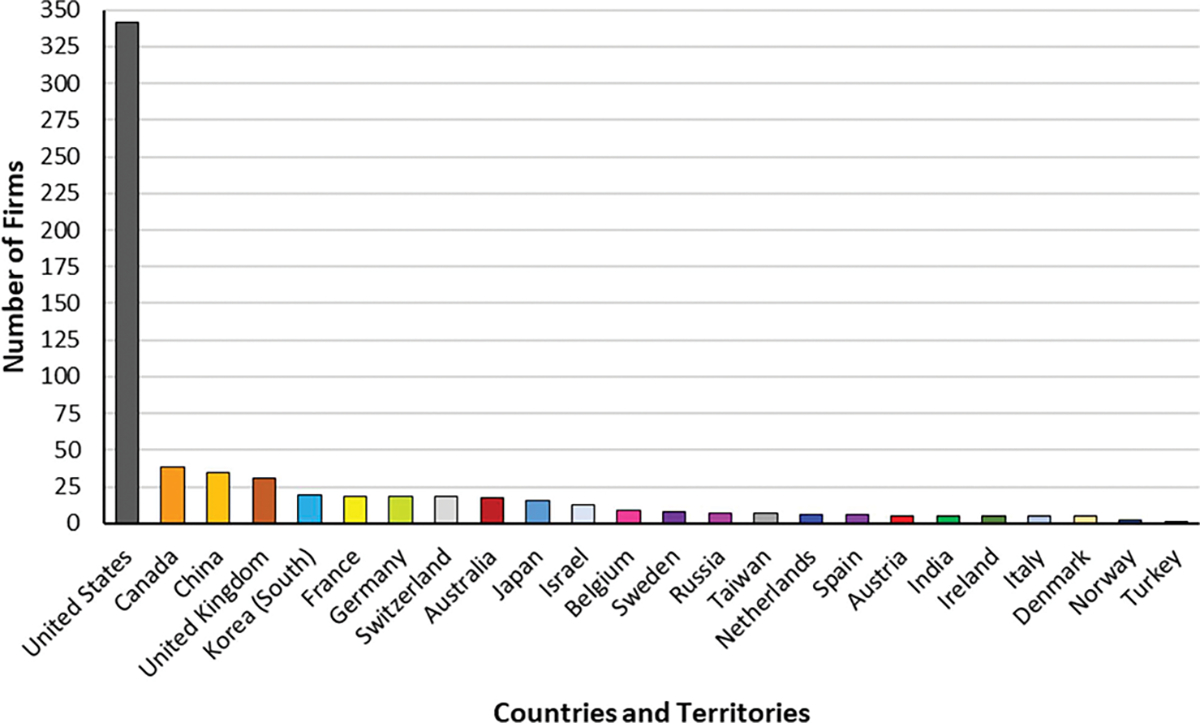
Geographic distribution of firms developing coronavirus treatments.

Our research investigates how firms innovate during a health crisis (i.e., coronavirus pandemic). We study the peak period of uncertainty before the first commercially available vaccine innovations came to market. This time window was between December 2019 and December 2020. In this context, we developed a novel cross-sectional dataset that includes 636 global biopharmaceutical firms from 24 countries and territories as illustrated in Fig. 2. Additional details about the dataset’s characteristics are displayed in Table II.

To build our dataset, we collected real-time product pipeline data from Biomedtracker for all companies developing treatments for COVID-19 (SARS-CoV-2). We follow prior studies that use firm product portfolios to explore product innovation (e.g., [15], [78]). This approach offers the potential for greater insights because it is challenging to access and compile a firm’s global product portfolio, especially within the context of the global biopharmaceutical industry, as the data are not often made publicly available in a timely manner. We used a systematic method to enhance the representativeness of our dataset by including 1) firms that developed radical innovations or repurposed innovations to treat coronavirus and 2) firms that were active in infectious disease research and could have developed innovations but chose not to do so. It was important to include all firm innovations during this time, which were at various stages of development. These included five broad categories: those in the discovery stage, those that had filed a new drug application, investigational new drug application (4) biologic license application, and clinical trial application with a federal regulator.

**Fig. 3.**
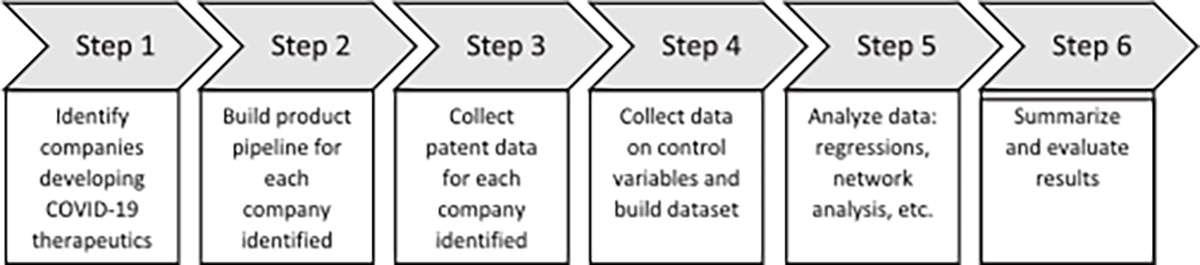
Research method diagram.

We also compiled the commercial, clinical, and regulatory activities of these companies by using data provided by the FDA’s monthly drug approval reports,^1^ the *Orange Book*,^2^ ClinicalTrials.gov, EU Clinical Trials Register, UMIN Clinical Trials Registry (Japan), Chinese Clinical Trial Registry, and Clinical Trials Registry—India. Patent data were obtained from Lens.org and additional company data were obtained from international stock exchanges and public sources. The process employed to collect and analyze the data is described in further detail in the research method diagram depicted in Fig. 3.

### B. Dependent Variables

Following prior studies that use product pipeline data to operationalize different types of innovations (e.g., [15], [78]), we draw upon a firm’s product portfolio to distinguish between two types of innovations: radical and repurposed. This approach is especially insightful in the context of the early stages of the COVID-19 pandemic because traditional approaches that rely on patent data to categorize types of innovations would not be appropriate given that patent applications are not published until 18-months after filing and the granting of a patent can take years. Hence, relying on patent-based measures alone would have precluded us from examining innovation in the early stages of the pandemic in a timely manner.

#### Radical Innovation:

An innovation was designated as being a *Radical Innovation* if its Biomedtracker disease indication was specifically “COVID-19” and its product ID did not overlap with that of any other product for any other disease, meaning that the product was not used to treat any other disease other than coronavirus. We measured *Radical Innovation* as a count of the number of new products a firm developed specifically to treat COVID-19.

#### Repurposed Innovation:

An innovation was designated as being a *Repurposed Innovation* if its Biomedtracker disease indication was specifically “COVID-19” and its product ID overlapped with that of one or more other products meant to treat any other disease. For instance, our dataset included drugs such as Abbvie’s application of Imbruvica, a therapeutic cancer drug, to treat COVID-19 and NasoVax, a monovalent intranasal influenza vaccine, to treat COVID-19. We measured *Repurposed Innovation* as a count of the number of existing products a firm repurposed to treat COVID-19.

### C. Independent Variables

#### Heterogeneous Knowledge (ln):

We followed prior studies (e.g., [15], [78]) by identifying all products in the focal firm’s product portfolio and the diseases they target. We then counted the number of unique disease categories (e.g., oncology, hematology, infectious diseases, etc.) and created our measure of *Heterogeneous Knowledge* based on the natural log transformed count of this quantity.

#### Homogeneous Knowledge (ln):

We identified all products in the focal firm’s product portfolio, similar to prior studies (e.g., [15], [78]), that targeted infectious diseases and created our measure of *Homogeneous Knowledge* based on the natural log transformed count of this quantity.

### D. Control Variables

We control for several observable factors that could influence innovation outcomes at the firm, product, and country levels. First, at the firm level, because larger firms tend to have greater resource endowments (e.g., cash, physical assets, human capital), we control for the scale and inertia in large firms by measuring *Firm Size* as the natural log transformed count of firm employees [79]. A firm’s knowledge stocks and capabilities evolve over time—likely influencing how the firm innovates. We calculate *Firm Age* as the natural log transformed number of years since the firm’s incorporation [79]. Due to potential differences in the resource endowments of public versus private firms, we control for whether a firm is publicly traded. We examined whether each firm was traded on any U.S. or foreign stock exchange to determine its public status. We measure *Public* as a binary variable that takes a value of 1 if the firm is publicly traded, and 0 otherwise [80]. Patents represent the codification of a firm’s accumulated stock of knowledge. Larger stocks of knowledge provide firms with a greater repository of information from which they may draw upon to create novel combinations of ideas [17], which can influence how a firm innovates. We measure *Knowledgebase* as the natural log transformed cumulative number of granted patents a firm possesses [81]. Additionally, since prior experience developing treatments for coronavirus may influence a firm’s decision to develop treatments for coronavirus, we control for *Prior Coronavirus Patents*, which represents the log transformed count of coronavirus patents granted to a focal firm.

Second, at the product level, we control for three different treatment technology categories. Using non-new molecular entities (non-NMEs) (i.e., existing marketed drugs) as our baseline for comparison, we control for whether the innovation is a *Biologic* (i.e., a large molecule drug derived from, or containing elements of, living organisms), *New Molecular Entity* (NME) (i.e., a new small molecule drug whose active ingredient is a chemical substance not previously marketed), or *Vaccine* (i.e., a preparation that bestows active immunity against a particular disease). Each of these three categories is measured as the natural log transformed count of the number of products each firm is developing in the specified category.

Finally, at the country level, we also control for governmental funding and economic status. We measure C*OVID-19 Funding* (millions of USD) as the natural log transformed amount of COVID-19 funding that each nation allocated to combat the pandemic. To account for potential differences between developed and emerging economies (e.g., institutions, resources, etc.), we include the dummy variable *Emerging Economy* that takes a value of one if the country is an emerging economy as defined by the United Nations World Economic Situation and Prospects report, and zero otherwise.

### E. Empirical Approach

In this study, we employ a Poisson regression estimation approach. Poisson regression is used to model nonnegative count data [82] and is frequently used in innovation studies (e.g., [83]). In cases where the dependent variable is a nonnegative count, such as in this study, ordinary least squares (OLS) linear regression models are not the most appropriate because the residuals will be heteroscedastic and nonnormal [84]. Taking the form of a log-linear model, the Poisson distribution assumes that the conditional mean of the outcome is equal to its variance [82]. This differs from the negative binomial model, which is a less restrictive generalization of the Poisson regression that is also used to model nonnegative count data [82], [84]. Negative binomial regression allows for overdispersion in the count data (i.e., the observed variance is greater than the mean) [84]. A likelihood ratio test confirmed that the data were not overdispersed and, consequently, the Poisson model was the preferred estimation approach for our data.

## IV. Results

We report descriptive statistics and pairwise correlations between variables in Table III. We calculated the variance inflation factor (VIF) and condition number to verify that multicollinearity was not a significant concern. The largest mean VIF value was 1.88 and the largest condition number was 4.86. The VIF and condition number are indicative of high multicollinearity when their values are greater than 10 and 30, respectively [85].

Table IV reports the results of the Poisson regression models. In Model 1, we specify the baseline model with only the control variables for *Radical Innovation*. In Model 2, we find that the coefficient for *Heterogeneous Knowledge (ln)* (*b* = −0.543, *p <* 0.01) is negative and significant, whereas the coefficient for *Homogeneous Knowledge (ln)* (*b* = 0.082, *p <* 0.05) is positive and significant. This suggests that, for a 1% change in a firm’s heterogeneous (or homogeneous) knowledge, the difference in the logs of expected counts of radical innovations is expected to be lower by 0.543 for heterogeneous knowledge, but higher by 0.082 for homogeneous knowledge, respectively. These results are easier to understand in terms of incidence rate ratios, which suggest that, for a 1% change in a firm’s heterogeneous (or homogeneous) knowledge, the rate of producing radical innovations is expected to be lower by a factor of 0.581 (e^−0.543^) for heterogeneous knowledge but higher by 1.085 (e^0.082^) for homogeneous knowledge. Hypothesis 1 suggests that firms with greater homogeneous knowledge will develop radical innovations to a greater extent than firms with heterogeneous knowledge. Therefore, we find support for hypothesis 1.

In Model 3, we specify the baseline model with only the control variables for *Repurposed Innovation*. In Model 4, we find that the coefficient for *Heterogeneous Knowledge* (*b* = 0.594, *p <* 0.01) is positive and significant and the coefficient for *Homogeneous Knowledge* (*b* = 0.066, *p* = n.s.) is positive but not significant. This suggests that, for a 1% change in a firm’s heterogeneous knowledge, the difference in the logs of expected counts of repurposed innovations is expected to be higher by 0.594. These results are easier to understand in terms of incidence rate ratios, which suggest that, for a 1% change in a firm’s heterogeneous knowledge, the rate of repurposing innovations is expected to be higher by a factor of 1.811 (e^0.594^). However, a firm’s homogeneous knowledge does not significantly impact repurposing innovations. Hypothesis 2 suggests that firms with greater heterogeneous knowledge will repurpose innovations to a greater extent than firms with homogeneous knowledge. Therefore, hypothesis 2 is supported.

### A. Robustness Checks

We test the sensitivity of the results to alternative specifications by performing a series of robustness checks. In a first series of tests, we log transformed the counts of *Radical Innovation* and *Repurposed Innovation* to correct for skewness in the data and performed several OLS regressions (see Table V). In a second series of tests, we performed several Poisson regressions that included an additional control variable, *R&D Spend*, that corresponds to the log transformed count of the focal firm’s R&D spending (in U.S. dollars) (see Table VI). Because data on R&D spending are primarily available only for publicly traded companies, the inclusion of this variable reduced the sample size to 284 observations. In a third series of tests, we performed several negative binomial regressions.^3^ The results of these tests were consistent with those of the original Poisson models.

### B. Post-Hoc Analysis

**Fig. 4.**
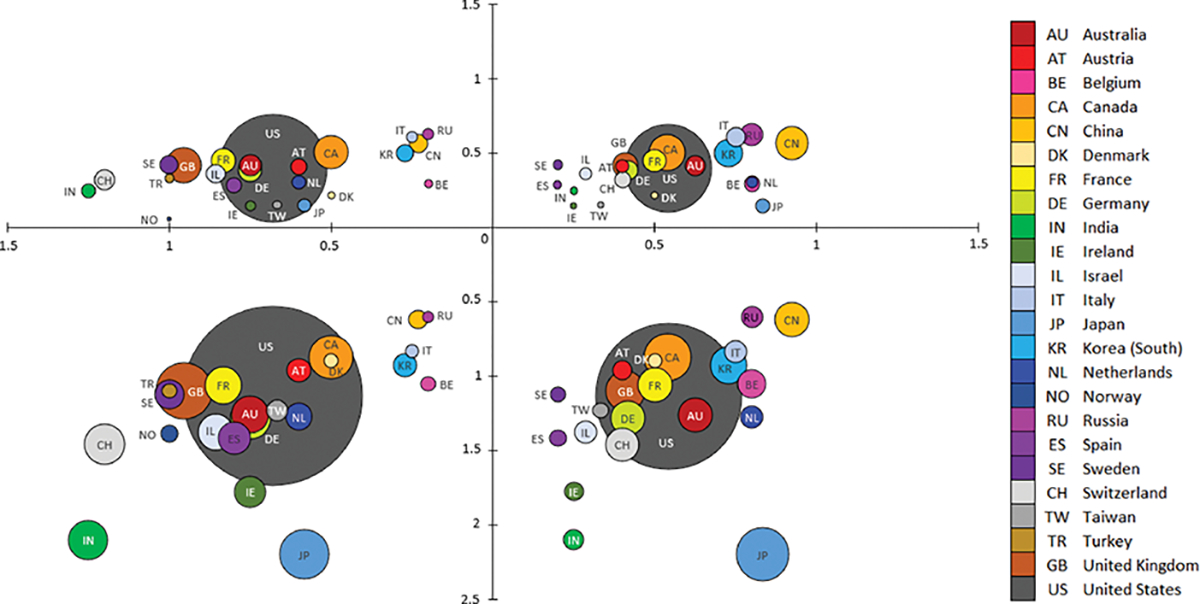
Firm experience and coronavirus innovations by a country/territory.

The richness of our unique dataset allowed us to explore how firms innovated during the coronavirus pandemic and how specific countries innovated during the early stages of this global crisis. Referring to Fig. 4, we aggregated the number of firms by country (i.e., the size of the circle represents the number of firms in each country). We plotted the relationship between knowledge attributes (heterogeneous versus homogeneous) and innovations (radical versus repurposed) into four quadrants (I–IV). Examining each quadrant reveals new insights into how different countries approached innovation during the pandemic.

In general, the United States had the most significant representation of firms in each quadrant (i.e., the largest circle). It tended to occupy a more central position within each quadrant’s cluster, which is consistent with prior research (e.g., [15]), size is not necessarily indicative of innovativeness. In terms of accumulated knowledge stocks, we find that firms have been heavily investing in heterogeneous knowledge at the expense of homogeneous knowledge. As Fig. 4 shows, the number of firms with deep knowledge of infectious diseases (I and II) tends to be fewer than those with a broader understanding of many different diseases (III and IV), as represented by the smaller circles. What is most striking from this analysis is that having a well-developed ecosystem within a country (i.e., the number of firms in a country) may not result in an innovation advantage in a crisis. We find that the average heterogeneous and homogeneous knowledge stocks of developed versus emerging economy countries are just as likely to be high versus low, suggesting a more competitive environment.

**Fig. 5.**
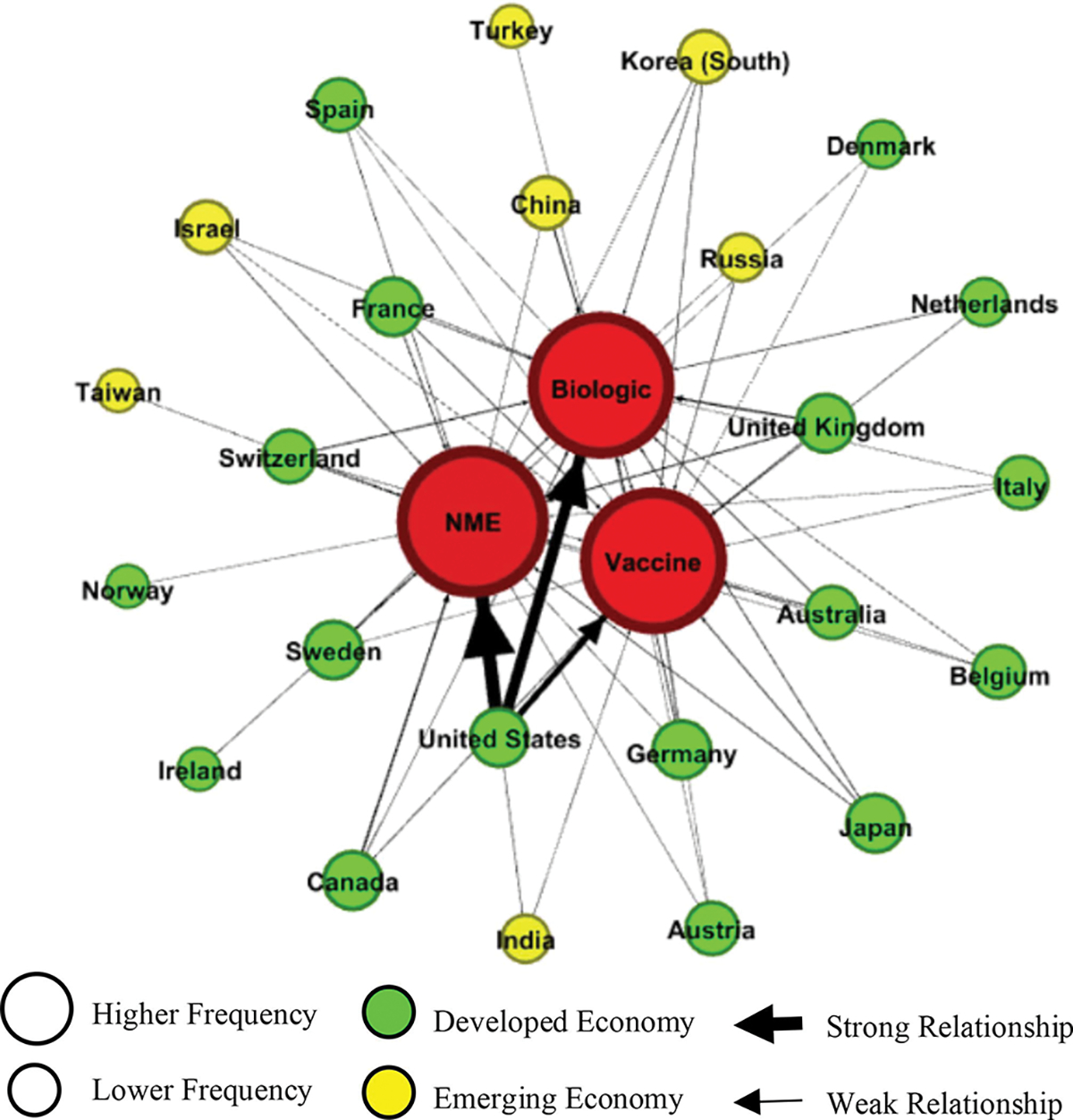
Relationships between countries/territories and the types of innovative treatments being developed for COVID-19.

To this end, we examined the relationships between various countries and the technologies they were developing to treat coronavirus. Fig. 5 illustrates a network analysis of these relationships. We found that, overall, there was nearly equal activity by countries to develop NMEs, biologics, and vaccines to treat coronavirus. Regarding technologies, the U.S. had a strong propensity toward developing NMEs (thickest arrow) and biologics to treat coronavirus, followed by vaccines. The network analysis revealed that developed economies (green circles), especially from the “Triad region” (i.e., the U.S., Western Europe, and Japan), were active in developing coronavirus treatments in all three technology categories and, thus, tended to be located closer to the center of the network. Developing economies (yellow circles) had a smaller representation and were typically relegated to the periphery of the network and were usually involved in developing coronavirus treatments in fewer technology categories.

## V. Discussion

The results of this study provide some interesting insights into how firms innovate in a crisis, such as the coronavirus pandemic. We discovered that firms with homogeneous knowledge developed more radical innovations than firms with greater heterogeneous knowledge. Conversely, we find that firms with greater knowledge diversity were more successful at repurposing innovations. These findings reveal the complexities that different contexts introduce into the innovation process that are often taken for granted. For the past several decades, firms have been following the conventional wisdom, based on the noncrisis literature, that they need to invest more in heterogeneous knowledge resources to improve the odds of creating radical innovations. Our research suggests that a unilateral movement away from homogeneous knowledge may have tipped the scales too heavily in favor of an overdiversification of therapeutic areas. Barring the next crisis, such strategies may be advantageous. But unfortunately, this crisis has taught us that a lack of investment in specialized knowledge may be detrimental if we face another crisis. Reversing this trend is a conscientious decision that is costly and requires a long-term commitment. Finding some degree of equilibrium is preferable, and crises may offer a window of opportunity in this regard.

We explored how countries innovated in the early stages of this crisis. Although most firms in our study resided in developed countries, they were no more likely than emerging economies to create radical and repurposed innovations. This discovery suggests that firms operating in emerging economies were provided more opportunities to innovate than expected. There were, however, distinctive intercountry innovation patterns. We found that countries that repurposed innovations more frequently (e.g., India, Switzerland) also tended to produce fewer radical innovations. Conversely, those countries that developed a more significant number of radical innovations on average (e.g., Japan, China) also tended to be less focused on repurposing innovations. Further, firms in emerging economies, such as Turkey, were focused exclusively on repurposing innovations.

Our analysis revealed that countries simultaneously approached this innovation challenge by developing different treatment technologies (e.g., NMEs, biologics, vaccines). While one would assume that there would be a preference for using one treatment over another (i.e., vaccines), this was not the case. Countries allocated resources similarly across treatment technologies. This finding is interesting since vaccines have often been the gold standard for combating viruses (e.g., polio vaccine). The initial uncertainty brought about by the exogenous crisis may have created strategic distortion (e.g., [86]), which prompted firms to consider alternative technologies that were faster to develop.

Our crisis-based research also highlights additional insights about firm competitiveness in high-tech industries. Interestingly, firm size was not a reliable predictor for radical or repurposed innovations. The pandemic created a window of opportunity that allowed smaller companies, with more limited resources, to compete with larger companies that possessed substantial resource endowments. Remarkably, older firms were no more likely to develop radical innovations or repurpose innovations than startups, which support the idea of a more level playing field in the early stages of the crisis. This result is interesting because older firms often possess more extensive product portfolios. So, it would make sense for them to attempt to repurpose existing products in their portfolio to accelerate market entry at a fraction of the cost of developing new treatments. Additionally, we found that the crisis reshaped the competitive environment, in which factors such as government funding and accumulated knowledge of prior diseases (i.e., coronavirus patents) had no impact on a firm’s innovative ability.

### A. Theoretical Implications

This study makes significant contributions to the extant literature. At its core, our study contributes to the innovation and crisis management literature works about how firms respond to an exogenous crisis through innovation. We expound our understanding about how crises may present a window of opportunity for firms that can recognize the shift in the uncertain, external environment, and act strategically to capitalize on the opportunity. The ability to see options during a crisis speaks to recent discussions in the literature about serendipity as a dynamic capability that, when harnessed, can play a significant role in an organization’s strategic response and subsequent performance outcomes [87], [88]. Early research on the topic equated serendipity with luck (i.e., being at the right place at the right time). More current research suggests that it involves having the right insights and resources to draw upon when approaching new opportunities [88].

It is important to note that serendipitous opportunities in and of themselves are not necessarily equally distributed across firms [89]. We attempt to add to this ongoing conversation by investigating how an organization’s prior knowledge resources (heterogeneous versus homogeneous knowledge) impacted not only its opportunity recognition but also how it was able to leverage such knowledge resources toward innovative endeavors (radical versus repurposed). Our study also contributes to the broader international business community and literature by revealing new insights about how firms from different countries approach an exogenous crisis through distinctive innovation strategies. Our study highlights that while pandemics are detrimental from a public health, economic, and education perspective, they also may create greater equality among new entrants and incumbents engaging in novel discoveries. This finding may indicate that crises afford emerging economies, often known as innovation laggards, a unique opportunity to compete on an equal footing with developed economies, and potentially leapfrog them.

### B. Practical Implications

This study has important implications for managers and policymakers alike. For managers, this study highlights the importance of building their expertise in infectious diseases since another health crisis is inevitable. Our results infer that firms with homogeneous knowledge of contagious diseases are better positioned to develop radical, innovative products and treatments to address the pandemic. As our findings indicate, the developers of innovative new vaccines and novel antiviral medications were the ones who captured the lion’s share of the market for new treatments. Consequently, firms with this capability may receive more research funding and grants. This finding may suggest that even though infectious diseases may not be as lucrative as other therapeutic areas (e.g., oncology treatments), managers should still pay close attention to this critical component of the firm’s knowledge management portfolio.

For policymakers, this study presents a cautionary warning. In an increasingly interconnected world, infectious diseases are not relegated to the confines of isolated villages on distant continents. The proliferation of contagious diseases is everyone’s problem. To combat the inevitable future pandemic, policymakers should direct more resources toward sponsoring collaborative R&D partnerships between government research institutes, the private sector, and universities (e.g., the NIH Accelerating COVID-19 Therapeutic Interventions and Vaccines and Rapid Acceleration of Diagnostics programs). As our results suggest, in the initial stages of understanding the new disease, the playing field was more level between nations. Thus, countries lagging in infectious disease research could initially compete by directing resources toward infectious disease research. Notwithstanding, this competitive landscape will only remain level for a short time. Policymakers should consider enacting programs to attract and retain talented researchers and invest in establishing the necessary laboratory infrastructure and protocols to conduct pathogenic research safely and responsibly in advance. Furthermore, as the results of this study indicate, policymakers may be better off allocating research funding and grants to those firms with deep expertise of infectious diseases, rather than those with more heterogeneous knowledge, if they aim to develop more novel treatments quickly and efficiently.

### C. Limitations and Future Research

This study also has some limitations that present opportunities for future studies. First, while we demonstrate how firms innovated in the face of a global health pandemic, it will be necessary for future researchers to examine the generalizability of our findings to other industries and other types of crises. Second, in this study, we examined how two organizational attributes, heterogeneous and homogeneous knowledge, influenced the innovation strategies of firms (i.e., develop new innovations or repurpose existing innovations). Second, in this study, we examined how two organizational attributes (i.e., heterogeneous versus homogeneous knowledge) influenced firms’ innovation strategies (i.e., radical versus repurposing). Future scholars could explore which types of innovations are preferable in a crisis scenario and which one results in higher product-market outcomes (e.g., market dominance, financial rewards, etc.). Importantly, the literature could benefit from a deeper understanding of the role that engaging in serendipitous opportunities created by exogenous crises play in shaping the organization’s future potential and associated outcomes (e.g., performance).

## VI. Conclusion

Crises challenge our conventional understanding, and the coronavirus pandemic is no exception. Given prior history, another pandemic was inevitable. However, we could not predict when or where it would emerge, which pathogen it would be or the extent of its virulence, its mode of transmission, its contagiousness, or how the public would respond. While some countries had previously developed response plans for an eventual pandemic, as Prussian Field Marshal von Moltke is credited with saying, “no plan survives first contact with the enemy.” Although no manner of preparation could have fully equipped us to mitigate the devastation and disruption that this crisis would unleash, this does not exonerate or excuse a lack of preparedness because the warning signs were evident. Years of declining investment in infectious disease research left the world less prepared as critical resources were diverted toward more financially lucrative diseases, such as cancer and cardiovascular disease. Notwithstanding, our study provides some encouraging results about the great strides that have been made in infectious disease research in such a short period. We hope that infectious disease research will soon garner the same attention that rare diseases have in recent years (e.g., [5], [8], [73]).

## Figures and Tables

**TABLE I:** Synthesis of Crisis-Based Research and Innovation

Novel Findings and Contributions	Selected References
Defined types of crises and fostered firm resilience strategies in response to current and future crises.	[23], [24], [29], [30], [38], [49]
Studied firm survival characteristics and repositioning costs in response to adverse events, industry shocks and periods of crisis.	[25], [26], [27]
Determined impact of economic and other crises on short- and long-term innovative investments across firms and nations.	[36], [40], [41], [42]
Examined entrepreneurial and innovation activities following the impact of natural disasters as well as other types of crises.	[28], [35], [56]
Utilized and developed business continuity innovation processes and patient centered business models to manage health crises	[31], [32]
Enhanced innovation management knowledge by addressing IP challenges using analytical techniques focused on developing a more robust response strategy to a crisis, especially early in the crisis.	[33], [57]
Studied differences in response strategies among actors in the innovation ecosystem (e.g., startups, mature firms, research institutions, family firms) during a crisis.	[44], [45]
Expanded open innovation processes and adopted flexible business model innovation strategies in a crisis.	[34], [90]
Re-examined the emerging crisis management literature on the benefits of repurposing innovation and engaging in frugal innovation in times of crisis.	[43], [74], [76]

**TABLE II:** Sample Characteristics

Characteristic	Details		%
*Innovation type*	Firms developing radical innovations		54.7
	Firms developing repurposed innovations	259	40.7
	Firms developing both radical and repurposed		4.6
*Company size (Number of employees)*	Less than 500		83.2
	500–5,000		8.6
	More than 5,000		8.2
*Company age (Years)*	Young (less than 20 years)		70.4
	Middle-aged (20–40 years)	143	22.5
	Mature (older than 40 years)		7.1
*Ownership type*	Publicly owned		54.1
	Privately owned		45.9
*Technologies under development*	New molecular entities		38.6
	Biologies		38.2
	Vaccines	162	23.2
*Countries*	Developed economies		70.8
	Emerging economies		29.2

**TABLE III:** Descriptive Statistics and Correlations

Variable	1	2	3	4	5	6	7	8	9	10	11	12	13	14

1. Radical Innovation	1.00													
2. Repurposed Innovation	**−0.51**	1.00												
3. Heterogeneous Knowledge (ln)	**−0.23**	**0.55**	1.00											
4. Homogeneous Knowledge (ln)	0.07	**0.38**	**0.62**	1.00										
5. Firm Size (ln)	0.02	**0.24**	**0.40**	**0.40**	1.00									
6. Firm Age (ln)	−0.06	**0.21**	**0.32**	**0.26**	**0.51**	1.00								
7. Public	−0.06	**0.23**	**0.40**	**0.27**	**0.27**	**034**	1.00							
8. Knowledgebase (ln)	−0.05	**0.30**	**0.44**	**0.39**	**0.52**	**0.48**	**038**	1.00						
9. Prior Coronavirus Patents (ln)	0.06	**0.09**	**0.18**	**0.33**	**032**	**034**	0.07	**037**	1.00					
10. COVID-19 Funding (ln)	0.00	0.05	0.06	**0.08**	**−0.08**	**−0.11**	0.00	0.00	0.06	1.00				
11. Biologic (ln)	**0.22**	**0.09**	0.06	0.04	**0.12**	0.04	−0.01	0.03	0.05	0.04	1.00			
12. New Molecular Entity (ln)	**−0.14**	**0.36**	**0.16**	**0.11**	−0.01	0.04	**0.12**	**0.14**	0.01	−0.02	**−0.45**	1.00		
13. Vaccine (ln)	**0.43**	**−0.24**	−0.06	**0.18**	**0.09**	0.07	−0.02	0.04	**0.11**	0.00	**−0.28**	**−034**	1.00	
14. Emerging Country	0.05	−0.11	**−0.10**	**−0.10**	**032**	**0.10**	0.04	−0.04	−0.06	**−0.29**	0.01	−0.06	0.05	1.00

Mean	0.68	0.50	0.80	0.79	4.19	2.50	0.54	2.22	0.28	12.78	0.27	0.29	0.17	0.14
Standard Deviation	0.76	0.63	0.82	0.86	2.38	0.98	0.50	2.16	0.76	3.03	0.37	0.36	0.32	0.34

Dependent variables denoted in gray.

Correlations in bold are significant at the *p* < 0.05 level

**TABLE IV:** Poisson Regression Results

	Model 1	Model 2	Model 3	Model 4

Variable	*Radical Innovation*		*Repurposed Innovation*	

Heterogeneous Knowledge (ln)		−0.543*** (0.057)		0.594*** (0.070)
Homogeneous Knowledge (ln)		0.082** (0.039)		0.066 (0.060)
Firm Size (ln)	−0.019 (0.017)	0.005 (0.017)	0.043** (0.021)	−0.036* (0.022)
Firm Age (ln)	−0.063 (0.040)	−0.040 (0.040)	0.047 (0.056)	0.025 (0.051)
Public	−0.125* (0.074)	0.044 (0.065)	0.304*** (0.102)	−0.010 (0.102)
Knowledgebase (ln)	−0.054** (0.022)	−0.006 (0.020)	0.071*** (0.022)	0.025 (0.023)
Prior Coronavirus Patents (ln)	0.047 (0.046)	0.029 (0.046)	−0.062 (0.055)	−0.069 (0.057)
COVID-19 Funding (ln)	−0.008 (0.011)	−0.004 (0.010)	0.019 (0.014)	0.011 (0.013)
Biologic (ln)	1.088*** (0.110)	1.144*** (0.068)	0.481*** (0.146)	0.382*** (0.138)
New Molecular Entity (ln)	0.522*** (0.148)	0.685*** (0.127)	1.036*** (0.160)	0.817*** (0.149)
Vaccine (ln)	1.572*** (0.083)	1.643*** (0.091)	−0.892*** (0.304)	−0.915*** (0.306)
Emerging Economy	0.183** (0.083)	0.066 (0.078)	−0.351** (0.163)	−0.108 (0.140)
Intercept	−0.750*** (0.181)	−0.890*** (0.178)	−1.972*** (0.249)	−1.768*** (0.236)

N	636	636	636	636

Pseudo R^2^	0.124	0.158	0.120	0.168

* *p* < 0.10

** *p* < 0.05

*** *p* < 0.01

Robust standard errors reported in parentheses.

**TABLE V:** OLS Regression Robustness Check

	Model 5	Model 6	Model 7	Model 8

Variable	*Radical Innovation (ln)*		*Repurposed Innovation (ln)*	

Heterogeneous Knowledge (ln)		−0.205*** (0.021)		0.211*** (0.020)
Homogeneous Knowledge (ln)		0.037* (0.019)		0.015 (0.020)
Firm Size (ln)	−0.005 (0.006)	0.008 (0.006)	0.018** (0.007)	−0.001 (0.007)
Firm Age (ln)	−0.033** (0.015)	−0.029** (0.014)	0.023 (0.017)	0.021 (0.015)
Public	−0.049* (0.028)	0.030 (0.027)	0.086*** (0.029)	−0.010 (0.027)
Knowledgebase (ln)	−0.019** (0.008)	−0.004 (0.007)	0.022*** (0.008)	0.004 (0.008)
Prior Coronavirus Patents (ln)	0.024 (0.018)	0.013 (0.019)	−0.019 (0.019)	−0.017 (0.019)
COVID-19 Funding (ln)	−0.006 (0.004)	−0.004 (0.004)	0.007 (0.005)	0.004 (0.004)
Biologic (ln)	0.485*** (0.064)	0.503*** (0.058)	0.130** (0.064)	0.088 (0.055)
New Molecular Entity (ln)	0.265*** (0.068)	0.303*** (0.062)	0.329*** (0.069)	0.258*** (0.060)
Vaccine (ln)	0.815*** (0.055)	0.780*** (0.057)	−0.183*** (0.064)	−0.189*** (0.061)
Emerging Economy	0.072* (0.038)	0.017 (0.034)	−0.111*** (0.040)	−0.039 (0.034)
Intercept	0.328*** (0.079)	0.299*** (0.076)	−0.058 (0.080)	−0.009 (0.073)

N	636	636	636	636

R^2^	0.349	0.451	0.266	0.415

* *p* < 0.10

** *p* < 0.05

*** *p* < 0.01

Robust standard errors reported in parentheses.

**TABLE VI:** Poisson Regression With R&D Spend Robustness Check

	Model 9	Model 10	Model 11	Model 12

Variable	*Radical Innovation*		*Repurposed Innovation*	

Heterogeneous Knowledge (ln)		−0.551*** (0.075)		0.522*** (0.080)
Homogeneous Knowledge (ln)		0.162*** (0.051)		−0.035 (0.066)
Firm Size (ln)	−0.050 (0.035)	−0.051 (0.033)	0.068** (0.030)	0.014 (0.030)
Firm Age (ln)	−0.063 (0.079)	0.029 (0.084)	0.006 (0.064)	−0.044 (0.058)
Public	−0.077 (0.194)	0.178 (0.312)	13.188*** (0.571)	14.197*** (0.643)
Knowledgebase (ln)	−0.086** (0.035)	−0.051 (0.034)	0.078*** (0.026)	0.045* (0.024)
Prior Coronavirus Patents (ln)	0.022 (0.072)	0.030 (0.076)	−0.018 (0.052)	−0.024 (0.055)
COVID-19 Funding (ln)	−0.003 (0.026)	0.003 (0.023)	0.007 (0.019)	−0.003 (0.017)
R&D Spend	0.069* (0.041)	0.091** (0.036)	−0.039 (0.031)	−0.048* (0.028)
Biologic (ln)	1.074*** (0.141)	1.026*** (0.089)	0.510*** (0.153)	0.501*** (0.134)
New Molecular Entity (ln)	0.412** (0.185)	0.457*** (0.174)	0.938*** (0.157)	0.832*** (0.146)
Vaccine (ln)	1.621*** (0.107)	1.669*** (0.122)	−0.600** (0.257)	−0.562** (0.280)
Emerging Economy	0.373** (0.157)	0.201 (0.154)	−0.622*** (0.224)	−0.361* (0.205)
Intercept	−1.824** (0.803)	−2.496*** (0.757)	−14.018*** (0.760)	−14.881*** (0.800)

N	284	284	284	284

Pseudo R^2^	0.192	0.233	0.128	0.167

* *p* < 0.10

** *p* < 0.05

*** *p* < 0.01

Robust standard errors reported in parentheses.

## Footnotes

1
https://www.accessdata.fda.gov/scripts/cder/daf/

2
https://www.accessdata.fda.gov/scripts/cder/ob/index.cfm

3 The results of these robustness tests are available upon request.
